# Carbohydrate-Binding Module and Linker Allow Cold Adaptation and Salt Tolerance of Maltopentaose-Forming Amylase From Marine Bacterium *Saccharophagus degradans* 2-40^*T*^

**DOI:** 10.3389/fmicb.2021.708480

**Published:** 2021-07-14

**Authors:** Ning Ding, Boyang Zhao, Xiaofeng Ban, Caiming Li, B. V. Venkataram Prasad, Zhengbiao Gu, Zhaofeng Li

**Affiliations:** ^1^State Key Laboratory of Food Science and Technology, Jiangnan University, Wuxi, China; ^2^School of Food Science and Technology, Jiangnan University, Wuxi, China; ^3^Collaborative Innovation Center for Food Safety and Quality Control, Jiangnan University, Wuxi, China; ^4^Department of Molecular Virology and Microbiology, Baylor College of Medicine, Houston, TX, United States; ^5^The Verna and Marrs McLean Department of Biochemistry and Molecular Biology, Baylor College of Medicine, Houston, TX, United States

**Keywords:** marine, cold adaptation, salt tolerance, maltopentaose, amylase, carbohydrate-binding module, linker

## Abstract

Marine extremophiles produce cold-adapted and/or salt-tolerant enzymes to survive in harsh conditions. These enzymes are naturally evolved with unique structural features that confer a high level of flexibility, solubility and substrate-binding ability compared to mesophilic and thermostable homologs. Here, we identified and characterized an amylase, SdG5A, from the marine bacterium *Saccharophagus degradans* 2-40^*T*^. We expressed the protein in *Bacillus subtilis* and found that the purified SdG5A enabled highly specific production of maltopentaose, an important health-promoting food and nutrition component. Notably, SdG5A exhibited outstanding cold adaptation and salt tolerance, retaining approximately 30 and 70% of its maximum activity at 4°C and in 3 M NaCl, respectively. It converted 68 and 83% of starch into maltooligosaccharides at 4 and 25°C, respectively, within 24 h, with 79% of the yield being the maltopentaose. By analyzing the structure of SdG5A, we found that the C-terminal carbohydrate-binding module (CBM) coupled with an extended linker, displayed a relatively high negative charge density and superior conformational flexibility compared to the whole protein and the catalytic domain. Consistent with our bioinformatics analysis, truncation of the linker-CBM region resulted in a significant loss in activities at low temperature and high salt concentration. This highlights the linker-CBM acting as the critical component for the protein to carry out its activity in biologically unfavorable condition. Together, our study indicated that these unique properties of SdG5A have great potential for both basic research and industrial applications in food, biology, and medical and pharmaceutical fields.

## Introduction

Maltooligosaccharides are important carbohydrate sources due to their ability to control glycemic response, regulate the immune system, and improve the colonic condition or gut microbiota in humans (Zhu et al., 2011; Nguyen and Haltrich, 2013; Chegeni and Hamaker, 2015; Huang et al., 2019). Structurally, they consist of 3–10 α-D-glucopyranosyl units linked by α-1,4 glycosidic bonds. Given their high water solubility, mild sweetness, and suitable viscosity, maltooligosaccharides are considered palatable and superior nutrient foods for the aged and infants (Pan et al., 2017). In particular, maltopentaose (G5), which contains five glucosyl units, is essential in medical and pharmaceutical fields for its use as a dietary nutrient for patients having renal failure or calorie deprivation and as a diagnostic reagent for the detection of α-amylase in serum and urine (Kandra, 2003; Wang et al., 2015). Therefore, the discovery of natural or engineered enzymes that can efficiently produce G5 is of great interest for both industrial and clinical settings.

However, microbial amylases known to specifically produce G5 are rare and the optimum temperatures of most maltopentaose-forming amylases (G5As) were found to be in the range of 60–93°C (Morgan and Priest, 1981; Hatada et al., 2006; Jana et al., 2013). Although the high temperature could increase reaction rates and mass transfer, and reduce viscosity, the processes are energy- and power-intensive and contribute significantly to CO_2_ emissions. Besides, high-temperature production might cause undesirable Maillard reaction, impair the physicochemical properties and nutritional value of maltooligosaccharides (Patel and Goyal, 2011). Hence, discovering G5As that enable efficiently producing G5 at low or room temperature is important. Interestingly, extremophiles, microorganisms that grow in extreme environmental conditions, were found to occasionally produce cold-adapted and/or salt-tolerant amylases evolved with unique structural features and physiological properties. These have provided economic and environmental advantages for their industrial applications (Sarmiento et al., 2015; Siddiqui, 2015; Santiago et al., 2016). For example, by using the cold-adapted amylases in the cleaning and detergent industry, a decrease of washing temperature from 40 to 30°C could reduce 30% electricity consumption and a further decrease of the temperature to 20°C has been expected to reduce CO_2_ emissions by half (Siddiqui, 2015). Moreover, salt-tolerant amylases have been applied to starch saccharification that used renewable, sustainable and economical marine microalgae as the substrate (Matsumoto et al., 2003; Karan et al., 2012).

Marine extremophiles and their secreted extremozymes including amylases have been of increasing interest because of their outstanding cold adaptability and salt tolerance (Liu et al., 2011; Qin et al., 2014; Jin et al., 2019; Zhu et al., 2020). *Saccharophagus degradans* 2-40^*T*^ (Sde 2-40) is a marine bacterium considered to be one of the most versatile polysaccharide-degrading organisms (Weiner et al., 2008). The complete genome of Sde 2-40 has been recently sequenced with the putative function of 128 genes encoding glycoside hydrolases being annotated (Weiner et al., 2008; Hutcheson et al., 2011). A distinct and common feature found in these enzymes is the wide presence of carbohydrate-binding module (CBM), suggesting the possible role of CBM on adapting to the marine environment and capturing the polysaccharides substrates in this highly dilute aqueous system (Weiner et al., 2008). As the CBM-containing enzymes from Sde 2-40 such as agarose, alginate lyase, xylanase, and glucanase have been successively studied to show their relatively high activities at low temperature and/or high salt concentration, we expect Sde 2-40 might be a potential producer of extremozymes (Kim et al., 2010, 2012; Ko et al., 2016; Wang et al., 2016).

In this study, we cloned a gene (*sdg5a*) encoding maltopentaose-forming amylase from Sde 2-40 (SdG5A) and expressed the recombinant SdG5A in *Bacillus subtilis*. SdG5A displayed strong product specificity of G5 with characteristics of cold adaptability, alkali stability, and salt tolerance. Further, these unique properties were examined to be attributed to the presence of CBM in SdG5A. Together, our findings provide valuable information for both basic and applied research concerning the discovery and molecular mechanisms of marine extremozymes as well as energy-efficient and economically profitable production of G5.

## Materials and Methods

### Bacterial Strains and Reagents

*Escherichia coli* JM109 and *B. subtilis* WB600 were used for recombinant DNA manipulations and protein production, respectively. Expression plasmid pST was obtained from laboratory stock. Maltooligosaccharides standards were purchased from Hayashibara Co., Ltd. (Okayama, Japan).

### Gene Cloning of *sdg5a* and *sdg5a-cad*

SdG5A was encoded by gene *sdg5a* with GenBank accession of AIV43244.1. The plasmid *sdg5a*/pST was constructed by amplifying the synthesized *sdg5a* gene (GENEWIZ, Suzhou, China) followed by In-Fusion cloning (Takara, Tokyo, Japan) with pST vector. To truncate the C-terminal region (Ile^428^-Phe^542^) of SdG5A and construct *sdg5a-cad*/pST, we first amplified the insert encoding the N-terminal region of SdG5A (Gln^1^-Ala^427^) with 20-bp homologous overlaps and pST vector simultaneously, and then assembled the two fractions by In-Fusion cloning. The assembled constructs *sdg5a*/pST and *sdg5a-cad*/pST were transformed into *E. coli* JM109 competent cells. Plasmids were extracted using QIAprep Spin Miniprep Kit (Qiagen, Shanghai, China) and transformed into *B. subtilis* WB600 competent cells for producing SdG5A and SdG5A-CAD, respectively.

### Expression and Purification of Recombinant SdG5A

Recombinant *B. subtilis* WB600 was inoculated into LB medium supplemented with kanamycin (50 μg mL^–1^) and grown at 37°C overnight in an orbital shaker at 220 rpm. The overnight culture was then inoculated into expression medium (36 g L^–1^ yeast extract, 0.17 M KH_2_PO_4_, 0.72 M K_2_HPO_4_, 10 g L^–1^ NaCl, pH 6.5) supplemented with kanamycin (50 μg mL^–1^) and incubated with shaking (220 rpm) at 25°C for 60 h. The extracellular crude enzymes were harvested by centrifugation at 10,000 × *g* for 30 min at 4°C.

Protein purification was performed using an ÄKTA purifier liquid chromatography system (Amersham Biosciences, Uppsala, Sweden). The supernatant containing crude protein was loaded onto a phenyl superose column (HR 10/10; Amersham Biosciences, Piscataway, NJ, United States) that had been pre-equilibrated with 20% (NH_4_)_2_SO_4_ in buffer A (20 mM phosphate buffer, pH 7.0). The column was eluted using distilled, deionized H_2_O at flow rate of 1 mL min^–1^. The active fractions were collected and loaded onto a Superdex 75 10/300 GL gel filtration column (GE Healthcare Biosciences AB, Uppsala, Sweden) that had been pre-equilibrated with buffer A, and then eluted with distilled, deionized H_2_O. Purified proteins were dialyzed against 10 mM phosphate buffer (pH 6.5) at 4°C for 24 h and determined on SDS-PAGE.

### Enzyme Activity Assay

SdG5A activity was measured by determining the reducing sugars released from the hydrolysis of starch using the 3,5-dinitrosalicylic acid (DNS) method (Miller, 1959). The reaction mixture containing 0.2 mL of appropriately diluted enzyme and 1.8 mL of 1.0% (w/v) soluble starch (Sinapharm Chemical Reagent, Shanghai, China) in 50 mM C_4_H_2_O_7_–Na_2_HPO_4_ buffer (pH 6.5), was incubated at 45°C for 15 min, quenched by adding 2.0 mL DNS reagent and boiled for 5 min. The absorbance was measured at 540 nm. One unit (U) of SdG5A activity was defined as the amount of enzyme that released reducing sugar equivalent to 1 μmol of glucose per minute under the assay conditions (45°C, pH 6.5).

### Determination of Hydrolysis Products

The reaction mixtures containing 0.2 mL of purified enzymes (2.0 U g^–1^ dry weight of starch) and 5.8 mL of corn starch (2%, w/w) were incubated at 4, 25, and 45°C in water bath for 4, 12, 24, and 36 h and boiled for 10 min to terminate the reaction. Samples were then centrifuged at 10,000 × *g* for 10 min and the supernatant was filtered through 0.22-μm syringe filter for high-performance anion-exchange chromatographic (HPAEC) analysis. The retention time and peak area of maltooligosaccharides standards (glucose – maltoheptaose) were compared with those of hydrolysis samples for identification and quantification.

Separations were carried out using an HPAEC system equipped with a pulsed amperometry detector (PAD; Dionex ICS-5000, Sunnyvale, CA, United States). Ten microliters of the sample solution was injected onto a CarboPacTM PA200 column (Dionex), and eluted with eluents A (0.25 M NaOH), B (1.0 M sodium acetate), and C (distilled, deionized H_2_O) at 30°C with a flow rate of 0.5 mL min^–1^ in a gradient elution program as follows: 0-6 min 8% A, 2% B, and 90% C; 6-6.5 min 8-12% A, 2-4% B, and 90-84% C; 6.5-18 min 12-24% A, 4-8% B, and 84-68% C; 18-25 min 24% A, 8-36% B, and 68-40% C; 25-40 min 24% A, 36% B, and 40% C; and 40-50 min 8% A, 2% B, and 90% C.

### Biochemical Characterization

The optimal pH for hydrolysis activity of purified SdG5A was determined at 45°C in 50 mM C_4_H_2_O_7_–Na_2_HPO_4_ buffer at a pH range of 2.5–8.0, and 50 mM glycine-NaOH buffer at a pH range of 8.0–12.0. The pH stability was determined by incubating the enzyme at varying pH values for 1 h at 4°C. The remaining activity was measured under the assay conditions (45°C, pH 6.5), and the activity before incubation was taken as 100%.

The optimal temperatures for hydrolysis activities of purified SdG5A and SdG5A-CAD were determined at temperatures ranging from 0 to 75°C in 50 mM C_4_H_2_O_7_–Na_2_HPO_4_ buffer (pH 6.5). The thermostability was determined by measuring the residual activities after incubation of the enzyme in C_4_H_2_O_7_–Na_2_HPO_4_ buffer (pH 6.5) at 25, 35, and 45°C for various periods. The remaining activity was measured under the assay conditions (45°C, pH 6.5), and the activity before incubation was taken as 100%.

To investigate the effects of different metal ions on the activity of SdG5A, activities were measured at 45°C and pH 6.5 with the addition of 50 mM of BaCl_2_, CaCl_2_, CuSO_4_, FeCl_3_, KCl, LiCl, MgSO_4_, NaCl, and ZnSO_4_. The salt tolerances of SdG5A and SdG5A-CAD were determined by measuring the residual activities after incubation of the enzyme in the presence of 0.1–4.0 M NaCl for 1 h at 4°C. The activity measured in the absence of any additive was taken as 100%.

### Structural Identity

Three-dimensional structures of SdG5A were predicted using the RaptorX template-based and distance-based protein structure modeling servers^1^ (Källberg et al., 2012; Xu, 2019). Quality of the predicted structure was evaluated using TM-score calculated by ResQ server^2^ (Yang et al., 2016). Estimation of residue-specific distance to the equilibrium native state was implemented by ResQ server (Yang et al., 2016). Multiple sequence alignments were performed using Clustal Omega^3^ (Sievers et al., 2011). Phylogenetic tree was constructed using the MEGA version 5.0 with the neighbor-joining algorithm (Tamura et al., 2014). Conserved domains were identified by the Conserved Domain Database^4^ (Marchler-Bauer et al., 2017). Solvent accessibility was predicted by ResQ server (Yang et al., 2016). Structures were rendered using the PyMOL version 2.2.3 software.

### Statistical Analysis

Three biologically independent replicates were used to calculate means and standard deviations. *P*-values were calculated using Prism 8 by performing two-tailed Student’s *t*-test, with a statistical significance level represented as ns (not significant), ^∗^*P* ≤ 0.05, ^∗∗^*P* ≤ 0.01, ^∗∗∗^*P* ≤ 0.001, and ^****^P ≤ 0.0001.

## Results

### Expression and Purification of SdG5A

**FIGURE 1:**
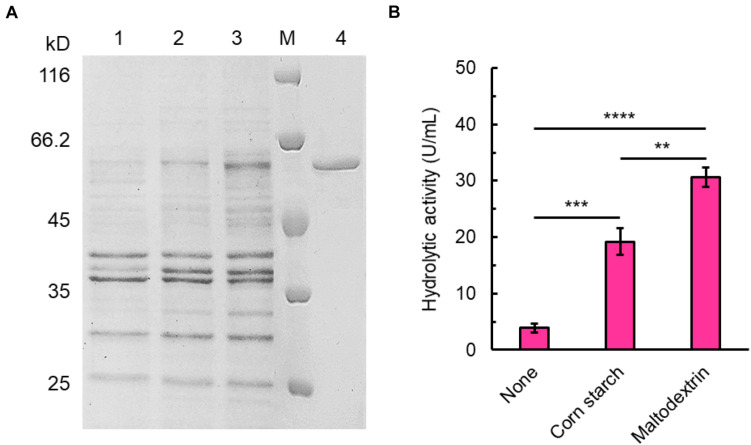
Expression and purification of SdG5A. **(A)** SDS-PAGE analysis of SdG5A. Lane M, molecular-weight protein marker. Lane 1, culture supernatant collected from the basic expression medium; lane 2, culture supernatant collected from the expression medium containing 5 g L^– 1^ corn starch; lane 3, culture supernatant collected from the expression medium containing 5 g L^– 1^ maltodextrin; lane 4, purified SdG5A. **(B)** Hydrolytic activity of culture supernatant collected from the mediums with the indicated supplement. Each value represents the mean of three independent measurements (mean ± standard derivation).

The open reading frame of SdG5A encodes a signal peptide of 21 amino acid residues at the N-terminus followed by mature protein of 542 amino acid residues, of which the calculated molecular mass was 60.06 kDa. Analysis of the open reading frame revealed the GC content of SdG5A to be 47.1%, which is similar to that of the Sde 2–40 whole genome (46.3%) and lower than maltooligosaccharide-forming amylases (MFAs) from other organisms (Weiner et al., 2008; Pan et al., 2017). We expressed SdG5A in the recombinant *B. subtilis* WB600 and purified SdG5A from the culture supernatant by the combination of phenyl superose and gel filtration column chromatography. The purity of SdG5A was confirmed by migration of the protein as a single band corresponding to a molecular mass of approximately 60 kDa on sodium dodecyl sulfate-polyacrylamide gel electrophoresis (SDS-PAGE) (Figure 1A). The specific activity of SdG5A was calculated to be 215 U mg^–1^. Furthermore, supplementing corn starch or maltodextrin in the basal medium dramatically increased the expression level of SdG5A (Figure 1A). The highest extracellular hydrolytic activity of SdG5A reached 30.6 U mL^–1^ when 5 g L^–1^ maltodextrin was added (Figure 1B).

### Production of Maltopentaose by SdG5A

**FIGURE 2:**
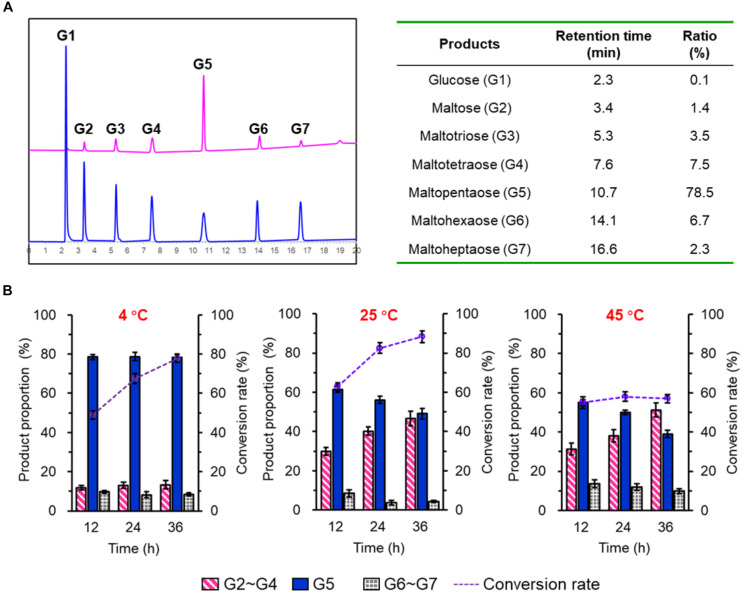
Product profile of purified SdG5A. **(A)** HPAEC-PAD chromatograms of maltooligosaccharides standards (blue) and hydrolysis products of corn starch by SdG5A (pink). Product proportions calculated from the chromatogram are listed in the table. Substrates (2%, w/w) were hydrolyzed by the purified enzyme (2.0 U g^– 1^ dry weight of starch) at 4°C for 4 h. **(B)** Product composition arising from the hydrolysis of corn starch by SdG5A at different temperatures. Substrates (10%) were hydrolyzed by the purified enzyme (2.0 U g^– 1^ dry weight of starch). Each value represents the mean of three independent measurements (mean ± standard derivation).

To investigate the product profiles of SdG5A, hydrolysates of corn starch were assayed using HPAEC-PAD (Figure 2A). At 4°C, we observed SdG5A converted 77.7% of the starch substrate within 36 h. Surprisingly, 78.5% of the total products were G5 while all other components showed the proportion below 10%. The product profiles were also tested at 25°C and the result showed higher reaction rates with the substrate conversion rates being 82.5 and 88.3% at 24- and 36-h hydrolyzation, respectively. However, SdG5A exhibited 1.4–1.6-fold lower product ratio for G5, compared to those generated at 4°C (Figure 2B). An increase of temperature up to 45°C caused a significant decrease in both the conversion rate and G5 production (Figure 2B), indicating that SdG5A might be more conductive to react at low temperature. The temperature effect on the G5 production by SdG5A suggested its potential to adapt cold and overcome the challenges of low molecular thermal motion and limited substrate accessibility in the cold environment.

### Cold Adaptation and Salt Tolerance of SdG5A

**FIGURE 3:**
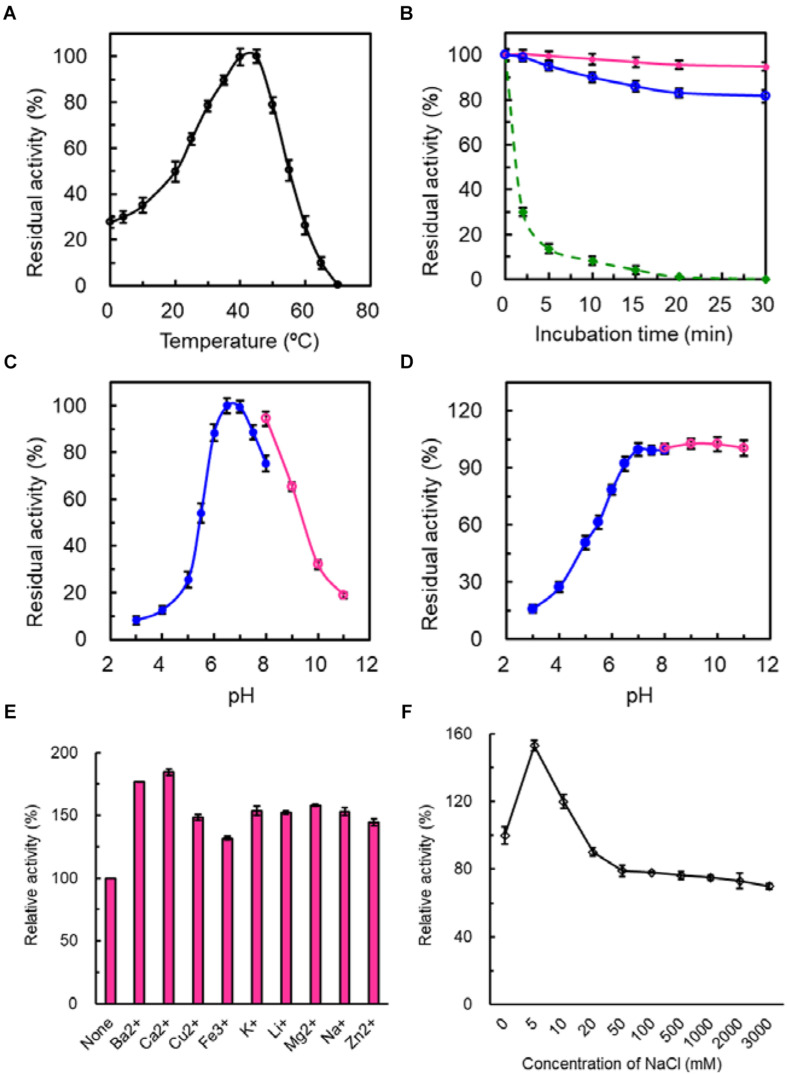
Enzymatic properties of purified SdG5A. **(A)** Effect of temperature on the catalytic activity. The temperature profile was determined in C_4_H_2_O_7_–Na_2_HPO_4_ buffer (pH 6.5) at different temperatures ranging from 0 to 70°C. The activity of the enzyme at 45°C was taken as 100%. **(B)** Effect of temperature on protein stability. To assess the thermostability, the enzyme was incubated at 25°C (pink), 35°C (blue), and 45°C (green). The residual activity was measured at pH 6.5 and 45°C. The activity before the incubation was taken as 100%. **(C)** Effect of pH on the catalytic activity. The pH profile was determined in 50 mM C_4_H_2_O_7_–Na_2_HPO_4_ buffer at pH range of 2.5–8.0 (blue) and 50 mM glycine-NaOH buffer at a pH range of 8.0–11.0 (pink) at 45°C. The maximum activity obtained at pH 6.5 was considered as 100%. **(D)** Effect of pH on protein stability. The pH stability was determined by incubating the enzyme in 50 mM C_4_H_2_O_7_–Na_2_HPO_4_ buffer at pH range of 2.5–8.0 (blue) and 50 mM glycine-NaOH buffer at pH range of 8.0–11.0 (pink) for 1 h at 4°C and the residual activity was measured at pH 6.5 and 45°C. The activity before the incubation was taken as 100%. **(E)** Effect of different metal ions on the catalytic activity. The activity of the enzyme with no addition was defined as 100%. **(F)** Effect of NaCl concentrations on the catalytic activity. The activity of the enzyme with no addition was defined as 100%. Each value represents the mean of three independent measurements (mean ± standard derivation).

The temperature for the highest catalytic activity of SdG5A was observed at 45°C and approximately 30% of its maximum activity was kept at 0 and 4°C, indicating its strong adaptation to the cold temperature (Figure 3A). In the absence of substrate, the purified SdG5A remained stable at 25 and 35°C for 30 min, retaining 95 and 80% of its maximal activity, respectively (Figure 3B). However, at 45°C the enzyme became heat-labile and the activity sharply decreased to 14% of the maximum within 5 min (Figure 3A). Although initially catalytically active at 45°C, SdG5A could also be deactivated within 20 min by incubating at the same temperature (Figure 3B). The purified SdG5A showed optimal activity within pH 6.5-7.0 (Figure 3C). Interestingly, no activity loss was observed even after the enzyme was incubated in the alkaline condition (pH 8.0-11.0) for 1 h without substrate added (Figure 3D). Additionally, the effects of various metal ions were studied under the optimal reaction condition (45°C, pH 6.5). We observed that several metal ions - Ba^2+^, Ca^2+^,Cu2^+^, Fe^3+^, K^+^, Li^2+^, Mg^2+^, Na^+^, and Zn^2+^ - could enhance the activity by 1.4-1.8-fold (Figure 3E). Moreover, the enzyme showed strong tolerance to the high salt concentration, retaining greater than 70% of its maximal activity in presence of 3 M NaCl (Figure 3F).

In sum, although SdG5A exhibited its maximum activity at the mesothermal optimum temperature and neutral optimum pH, in case of the extremely harsh reaction condition it might manifest its naturally adapted molecular mechanisms to maintain the persistent activity.

### Structure Analysis of SdG5A

**FIGURE 4:**
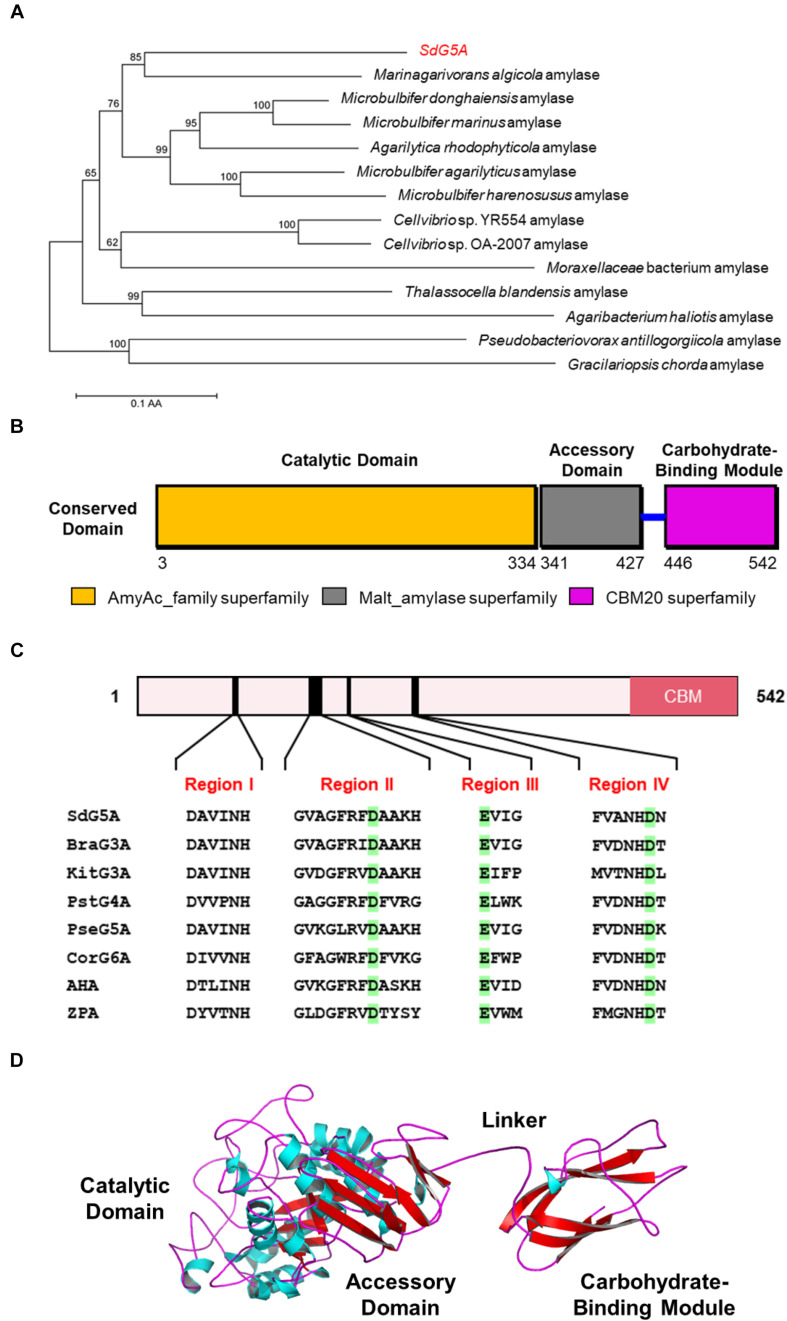
Sequence and three-dimensional structure analysis of SdG5A. **(A)** Phylogenetic tree of the amino acid sequences of SdG5A. The tree was constructed using the neighbor-joining algorithm of the MEGA program (version 5.0). Bootstrap values (*n* = 1,000 replicates) are reported as percentages. The scale bar represents the number of changes per amino acid position. **(B)** Analysis of the conserved domains identified by Conserved Domain Database (Marchler-Bauer et al., 2017). **(C)** Conserved regions I–IV of α-amylases. Amino acid residues corresponding to catalysis are highlighted in green. **(D)** Template-based predicted structure model of SdG5A.

To understand the potential mechanisms that might underlie the cold adaptation and salt tolerance of SdG5A, we conducted a comprehensive *in silico* structural analysis. The phylogenetic tree provides a visual representation of the evolutionary interrelations of proteins derived from a common ancestral (Saitou and Nei, 1987). Our phylogenetic analysis revealed that SdG5A is closely related to the putative amylase from the marine bacteria *Marinagarivorans algicola* (GenBank accession WP_053982064) with a 67% sequence identity (Figure 4A), but has low identities (15-44%) with other MFAs (data not shown). We then analyzed the conserved domains to find specific structural and functional units in SdG5A that may have given rise to its special properties during the molecular evolution. The conserved domain footprints of SdG5A were identified by searching in Conserved Domain Database, a collection of annotated multiple sequence alignment models (Marchler-Bauer et al., 2017). We found the modular structure of SdG5A comprises mainly three domains - Domain of AmyAc_family superfamily (AmyAc, Arg^3^ to Thr^334^), Domain of Malt_amylase superfamily (Malt, Thr^341^ to Ala^427^), and CBM20 (Val^446^ to Phe^542^) (Figure 4B). Particularly, AmyAc and CBM20 located at the two terminals of SdG5A are accountable for catalysis and substrate binding, respectively. The two domains are separated by the accessory module Malt and an extended linker (Ile^428^ to Lys^445^).

To characterize the extent of homology of SdG5A compared to other reported enzymes, we selected two other cold-adapted and salt-tolerant α-amylases from *Alteromonas haloplanctis* (AHA) and *Zunongwangia profunda* (ZPA) (Feller et al., 1992; Qin et al., 2014). At the same time, we selected five other non-extremophilic MFAs, including maltotriose-forming amylase from *Brachybacterium* sp. LB25 (BraG3A) and *Kitasatospora* sp. MK-1785 (KitG3A), maltotetraose-forming amylase from *Pseudomonas stutzeri* MO-19 (PstG4A), maltopentaose-forming amylase from *Pseudomonas* sp. KO-8940 (PseG5A) and maltohexaose-forming amylase from *Corallococcus* sp. EGB (CorG6A) for comparison (Table 1; Fujita et al., 1989; Shida et al., 1992; Doukyu et al., 2007; Kamon et al., 2015; Li et al., 2015). Four conserved sequence regions (Region I to IV) that had been discovered and used to define α-amylases in glycosyl hydrolase family 13 were found in all 8 amylase targets (Figure 4C; Janeček, 2002). Specifically, three conserved catalytic residues corresponding to Asp^163^, Glu^189^, and Asp^254^ in SdG5A were invariantly conserved throughout the MFAs, AHA, and ZPA (Figure 4C). It should be noted that all six MFAs contain multiple domains including a CBM20, which, however, is absent from AHA and ZPA.

**TABLE 1:** Information of the enzymes used for the comparison with SdG5A in this study.

Name	Enzyme	Source	GenBank accession^*a*^	References
AHA	α-Amylase	*Alteromonas haloplanctis*	P29957.3	Feller et al., 1992
ZPA	α-Amylase	*Zunongwangia profunda*	WP_013072233.1	Qin et al., 2014
Aga50D	β-Agarase	*Saccharophagus degradans* 2-40^*T*^	4BQ4	Kim et al., 2010
Xyn10C	β-Glycosidase	*Saccharophagus degradans* 2-40^*T*^	ABD82280.1	Ko et al., 2016
BraG3A	G3A	*Brachybacterium* sp. LB25	BAE94180.1	Doukyu et al., 2007
KitG3A	G3A	*Kitasatospora* sp. MK-1785	HW429890.1	Kamon et al., 2015
PstG4A	G4A	*Pseudomonas stutzeri* MO-19	AAA25707.1	Fujita et al., 1989
PseG5A	G5A	*Pseudomonas* sp. KO-8940	BAA01600.1	Shida et al., 1992
CorG6A	G6A	*Corallococcus* sp. EGB	AII00648.1	Li et al., 2015

*^a^Accession numbers deposited in the National Center for Biotechnology Information (NCBI) database (https://www.ncbi.nlm.nih.gov/). G3A, maltotriose-forming amylase; G4A, maltotetraose-forming amylase; G5A, maltopentaose-forming amylase; G6A, maltohexaose-forming amylase.*

We then implemented a template-based structural prediction of SdG5A using the RaptorX web server (Figure 4D; Källberg et al., 2012). The domains for AmyAc and Malt were simulated with the crystal structure of amylase from *Pseudoalteromonas haloplanktis* as template (PDB entry: 1AQH, 50.2% identity to SdG5A), while the domain for CBM20 was modeled based on the structure of cyclodextrin glucanotransferase from alkalophilic *Bacillus* sp. (PDB entry: 1I75, 35.5% identity to SdG5A). The overall structure showed high fitted score with the TM-score of 0.59 (TM-score ranging from 0.5 to 1.0 indicated the same fold with template). The catalytic domain adopted the structure of a (β/α)_8_-barrel formed by eight parallel β-strands surrounded by eight α-helices. At the opposite side of this catalytic domain, the CBM20 domain folded as an antiparallel β-barrel structure. As expected, the accessory module, which consisted only of β-sheets followed by a flexible linker, separated the N-terminal catalytic domain and C-terminal CBM20 to allow their independent movements toward and against each other (Figure 4D). Together, our general structural analysis established the enzymatic key players of SdG5A and provided possible indicators giving rise to its persistent function in the cold or high salt setting.

### Structural Determinants of Cold Adaptation and Salt Tolerance

#### Distribution of Charged Amino Acid Residues

**FIGURE 5:**
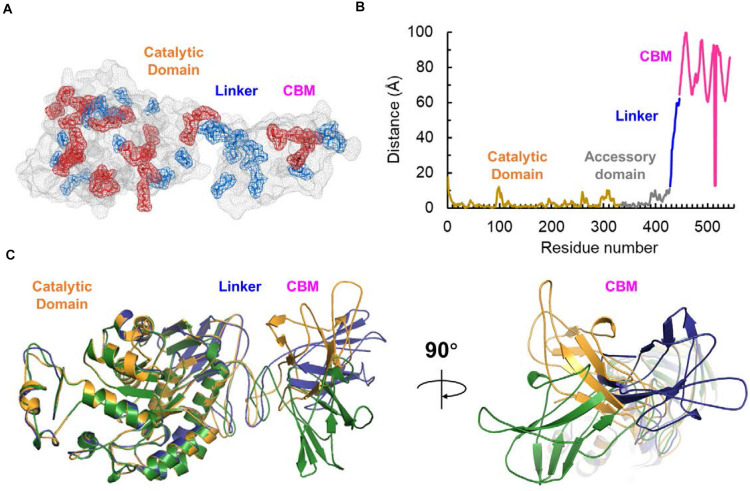
Surface electrostatic potential and flexibility analysis of SdG5A. **(A)** Distribution of charged residues on the SdG5A surface. Acidic residues including glutamic acid and aspartic acid are indicated in blue; basic residues including histidine, lysine, and arginine are indicated in red. **(B)** Residue-specific distance to the equilibrium native state. The distance was estimated by ResQ server based on the support vector regressions. **(C)** Distance-based predicted structural model of SdG5A. All structures were predicted using the RaptorX web server and rendered using the PyMOL Version 2.2.3 software.

Bioinformatic studies have shown that a higher ratio of acidic (glutamic acid and aspartic acid) to basic (histidine, lysine, and arginine) residues is commonly observed from the cold-adapted and salt-tolerant enzymes. We analyzed the composition of amino acid residues for the different domains in SdG5A (Supplementary Table 1) and compared the frequencies of charged residues in SdG5A and each domain with those in single-domain AHA and ZPA (Table 2). Initially, the proportion of acidic residues and basic residues in SdG5A as a whole were similar, inconsistent with the postulation that acidic amino acid residues are dominant in the cold-adapted and salt-tolerant enzymes. However, different results were observed when we calculated the proportion for each domain separately. The proportion of acidic residues in the linker and CBM region was 22.2 and 4.1% higher than that of basic residues, respectively. The net charge densities were higher than that of AHA and ZPA, which possessed 2.8 and 3.7% excess acidic residues, respectively (Table 2). In contrast, for the catalytic domain and accessory domain, basic residues were found in excess in SdG5A (Table 2). This difference in the distribution of charged residues was visually represented in Figure 5A. In particular, numerous acidic amino acid residues being widespread along the trailing end of the linker was indicative to generate strong electrostatic repulsions between the neighboring residues and accommodate conditions for the CBM domain to have greater access to solvent and substrates in an unfavorable cold and hypersaline condition.

**TABLE 2:** Comparison of the frequencies of charged amino acid residues for different domains in SdG5A and other extremophilic amylases.

Parameter	SdG5A					AHA	ZPA
	Catalytic domain	Accessory domain	Linker	CBM	Entire protein		
Acidic residues (%)	9.9	7.5	27.8	9.3	10.0	10.2	15.0
Basic residues (%)	11.7	8.6	5.6	5.2	9.8	7.8	11.8
Net charge density (*z*, %)^*a*^	1.6	1.1	−22.2	−4.1	−0.2	−2.8	−3.7

*^a^Net charge density is estimated by z = (n_basic_ − n_acidic_)/length × 100%, where n_basic_ and n_acidic_ are the number of basic and acidic residues, respectively. Acidic residues include glutamic acid and aspartic acid. Basic residues include histidine, lysine, and arginine. All sequences are presented in the Supplementary Material. AHA, α-amylase from Alteromonas haloplanctis; ZPA, α-amylase from Zunongwangia profunda.*

#### Protein Flexibility

One postulation of cold adaptation is that proteins require high conformational flexibility. To characterize the global flexibility of SdG5A, we used ResQ to estimate the distance to which each residue can travel from the same residue at its native state, at which the protein atoms stay at the equilibrium position in the lowest free energy conformation (Yang et al., 2016). Across the residues of SdG5A, these distances stayed low and smooth for the catalytic domain but high and unsteady for the linker-CBM region, indicating a variety of conformational states the linker-CBM region of SdG5A could cover (Figure 5B). We further used a distance-based modeling based on the convolutional residual neural network to characterize the contact between the catalytic and linker-CBM region (Xu, 2019). Through the multiple models fitting of the prediction parameters, we observed that although the catalytic domain displayed similar folds and positions in different models, the linker could adopt a wide range of conformations, providing enough occurrences to stretch and bend and promoting contacts between the catalytic domain and CBM (Figure 5C).

Together, these findings indicated that the cold adaption and salt tolerance of SdG5A could largely be governed by the non-catalytic linker-CBM region having enhanced conformational mobility and flexibility compared to the catalytic domain.

### Truncation of the Linker-CBM Region in SdG5A

**FIGURE 6:**
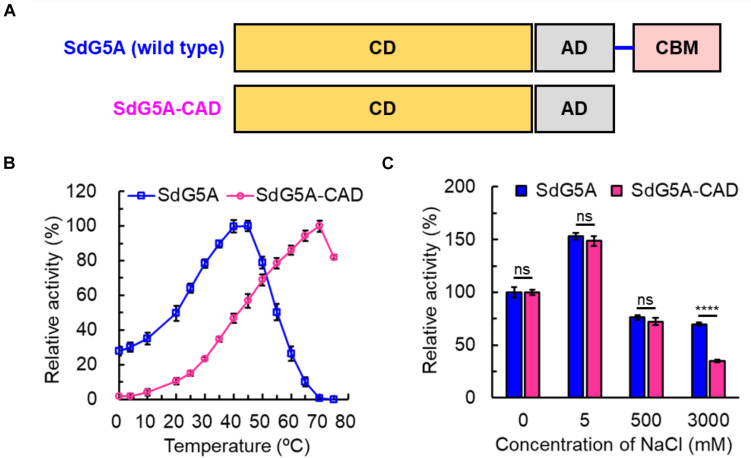
Characterization of SdG5A-CAD. **(A)** Schematic of truncation of the linker-CBM region of SdG5A. **(B)** Effect of temperature on the activity. The temperature profile was determined in C_4_H_2_O_7_–Na_2_HPO_4_ buffer (pH 6.5) at different temperatures ranging from 0 to 75°C. **(C)** Effect of NaCl concentrations on the activity. The activity of the enzyme with no addition was defined as 100%. Each value represents the mean of three independent measurements (mean ± standard derivation).

To validate our bioinformatics analysis and further investigate the role of the linker-CBM region of SdG5A, we truncated the region of Ile^428^-Phe^542^ and preserved the catalytic domain and accessory module to construct SdG5A-CAD (Figure 6A). SdG5A-CAD was expressed in *B. subtilis* WB600 and purified using phenyl superose and gel filtration column chromatography. Compared with the data of enzymatic properties observed from the full-length SdG5A, removal of the linker-CBM region resulted in approximately 30.2-fold reduction of the activity at 0°C (2.0 U mg^–1^), and 2.0-fold reduction of the relative activity in 3 M NaCl (34.9%), suggesting that the linker-CBM region played a critical role for providing its cold adaptation and salt tolerance behavior (Figures 6B,C). These experimental results were consistent with our previous bioinformatics analysis and successfully demonstrated that SdG5A is a cold-adapted and salt-tolerant enzyme driven by its highly flexible, negatively charged and functionally distinct substrate-targeting linker-CBM region for the efficient G5 production.

## Discussion

Global energy consumption and warming are rapidly increasing due to industrial activities and advances. Food system was estimated to contribute 34% of all human-made CO_2_ emissions (Crippa et al., 2021). Thus, the application of cold-adapted enzymes has great potential for large-scale industrial food biocatalytic processes due to the avoidance of heating reactors, which results in saving of energy consumption and CO_2_ emissions. At the same time, the utilization of cold-adapted enzymes in food processing could improve the quality of food by minimizing undesirable chemical reactions, retaining volatile flavor compounds and preventing modification of heat-sensitive substrates and products occurring at high temperature during enzymatic reaction or inactivation (Sarmiento et al., 2015; Siddiqui, 2015; Santiago et al., 2016). It should be noted that G5, as one of the functional oligosaccharides, tends to suffer nutrition loss and changes in physicochemical properties at high temperature (Patel and Goyal, 2011). Therefore, compared to mesophilic or thermostable G5As, of which the optimum temperatures range between 60 and 93°C, SdG5A has greater prospects to be used in energy-efficient and environmentally friendly industrial production of G5. At room temperature, SdG5A could convert more than 80% of the corn starch into maltooligosaccharides with the yield of approximately 60% of G5 within 24 h. Among the previously reported G5As, the one secreted from *Bacillus megaterium* VUMB109 showed the highest efficacy for producing G5 at 90°C, with the conversion rate of corn starch and the product specificity being approximately 20 and 40%, respectively (Supplementary Table 2; Jana et al., 2013). In comparison, without intensive heating, SdG5A was able to increasing the G5 yield by approximately sixfold.

Another unique characteristic of SdG5A is the ability to tolerate high salt concentration. In recent years there has been an increasing interest in using microalgae to produce fermentable sugar (Matsumoto et al., 2003; Ma et al., 2020). Marine microalgae are superior to land crops as feedstocks for bioconversion because they are capable of converting solar energy and CO_2_ to starch through photosynthesis (Khan et al., 2018). However, the utilization of marine microalgae as substrate for conventional amylases required desalinization, which led to additional cost (Matsumoto et al., 2003). Hence, the salt-tolerant SdG5A would be a promising candidate for the production of maltooligosaccharides with the abundant and renewable marine microalgae.

**FIGURE 7:**
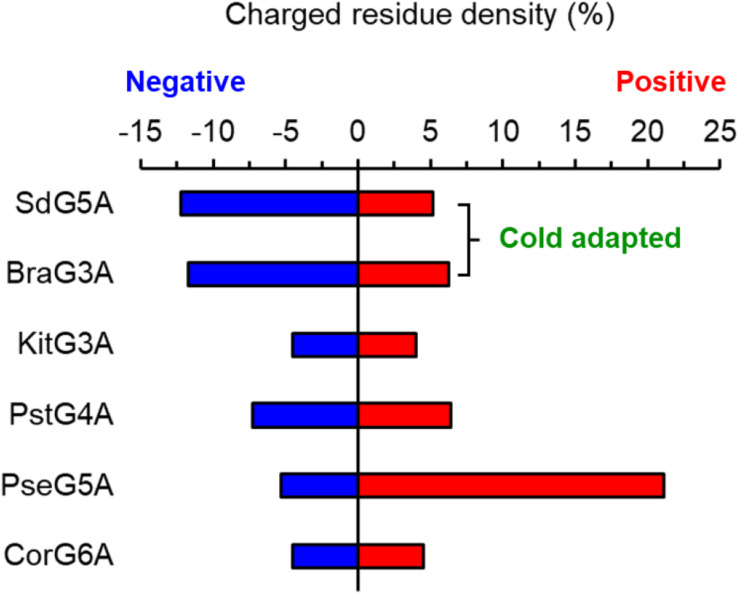
Density of charged amino acid residues in the linker-CBM region of maltooligosaccharide-forming amylases.

To date, there have been only two α-amylases found to be concurrently cold-adapted and salt-tolerant: amylase from Antarctic bacterium *Alteromonas haloplanctis* (AHA) and amylase from marine bacterium *Zunongwangia profunda* (ZPA) (Feller et al., 1992; Srimathi et al., 2007; Qin et al., 2014). Although the optimal temperatures of AHA and ZPA were lower than SdG5A by 10–20°C, the relative activities at 0°C for the three enzymes were comparable. In addition, compared to AHA and ZPA, which retained 80 and 74% of the initial activity at 4.5 and 4.0 M NaCl, respectively (the activity of the enzymes with no addition was defined as 100%), SdG5A showed relatively weaker salt tolerance, but significantly stronger salt tolerance compared to other reported G5As (Supplementary Table 2; Jana et al., 2013). With regard to the structural features that conferred cold adaptation and salt tolerance, all three amylases possessed high percentage of acidic residues and high conformational flexibility in entire or partial protein structure. In general, cold and high salinity affect properties and structures of enzymes by restricting the availability of free water molecules for protein hydration (Karan et al., 2012). Therefore, improved characteristics are critical for extremozymes to prevent self-aggregation when water is scarce from their surfaces. The high density of identically charged residues promotes weak but appreciable repulsive protein–protein interactions, thus promoting molecular motion. Considering the contribution of charged residues on solubility, acidic residues are superior to basic residues due to their less hydrophobic solvent-accessible area of the side-chain components. Besides, the negatively charged residues bind to hydrated cations and form a hydration layer on the protein surface, thereby reducing their surface hydrophobicity (Karan et al., 2012). As a result of the improved solubility and flexibility, AHA, ZPA and SdG5A exhibited low aggregation and high activity at extremely temperatures and high salt concentrations. However, different from the single-domain amylases AHA and ZPA, SdG5A was composed of three domains and one extended linker. Instead of possessing an evolved overall structure, SdG5A only relied on the negative surface potential and high flexibility of the linker-CBM region to maintain the cold adaptation and salt tolerance. Consistent to our *in silico* analysis, truncation of the linker-CBM region resulted in a significant decline in the activities at low temperature and high salt concentration, indicating the linker-CBM acted as a critical component that endowed these unique properties to the protein to carry out its activity even in the biologically unfavorable condition. As for other multi-domain MFAs, mesophilic KitG3A, PstG4A, PseG5A, and CorG6A possessed equivalent acidic and basic amino acid residues or higher percentage of basic residues in the linker-CBM region, while only BraG3A followed the same trend as SdG5A (Figure 7). Although BraG3A has not been clearly defined as a cold-adapted enzyme, we note that a study has shown that it still had higher cold adaptivity than other MFAs, retaining approximately 25% of its maximal activity at 10°C (Doukyu et al., 2007). This reflects a close relationship between the linker-CBM region and cold adaptivity in MFAs. Furthermore, it is interesting to note that cysteine contributes to the highest ratio (22.2%) of the total amino acid residues in the linker region of SdG5A (Supplementary Table 1). This is contrary to the fact that cysteine residues are often buried and known to be the least abundant to get exposed on the protein surfaces during evolution due to its hydrophobicity (Marino and Gladyshev, 2010). The presence of exposed cysteines in the SdG5A linker highlights its unique functional behavior and adaptation which might be due to the intrinsic reactivity of cysteine residues affected by its large p*K*_*a*_ fluctuations to confer more protein flexibility (Marino, 2014).

Marine microbial enzymes have presented a promising outlook for both applied and basic research. Sde 2-40 was emerging as a vanguard of marine bacteria and functioning as a “super-degrader” of complex carbohydrates (Weiner et al., 2008). Like SdG5A, many of the enzymes from Sde 2-40 exhibited outstanding cold adaptation and architecture comprising the joint catalytic domain and CBM. However, the relationship between the unique properties and structure has not been adequately studied (Kim et al., 2010; Ko et al., 2016). Hence, we analyzed the frequencies of charged residues for CBM in the cold-adapted β-agarase (Aga50D) and β-glycosidase (Xyn10C) from Sde 2-40 (Table 3). Interestingly, both of the enzymes possess abundant excess acidic residues in their CBMs, which is similar to SdG5A. Therefore, it could be postulated that the role of CBM on protein adapting cold is also applicable to more carbohydrases in Sde 2-40 or other marine bacteria.

**TABLE 3:** Properties of the cold-adapted enzymes from Sde 2-40 and the frequencies of charged amino acid residues for CBM.

Enzyme	SdG5A	Aga50D	Xyn10C
Properties	Retain ∼30% of its maximum activity at 4°C	Retain ∼78% of its maximum activity at 20°C	Retain ∼80% of its maximum activity at 20°C
Acidic residues (%)	9.3	12.9	13.9
Basic residues (%)	5.2	9.3	4.9
Net charge density (*z*, %)^*a*^	−4.1	−3.6	−9.0

*^a^Net charge density is estimated by z = (n_basic_ - n_acidic_)/length × 100%, where n_basic_ and n_acidic_ are the number of basic and acidic residues, respectively. Acidic residues include glutamic acid and aspartic acid. Basic residues include histidine, lysine, and arginine. All sequences are presented in the Supplementary Material. Aga50D, β-agarase from Saccharophagus degradans 2-40^T^; Xyn10C, β-glycosidase from Saccharophagus degradans 2-40^T^.*

**FIGURE 8:**
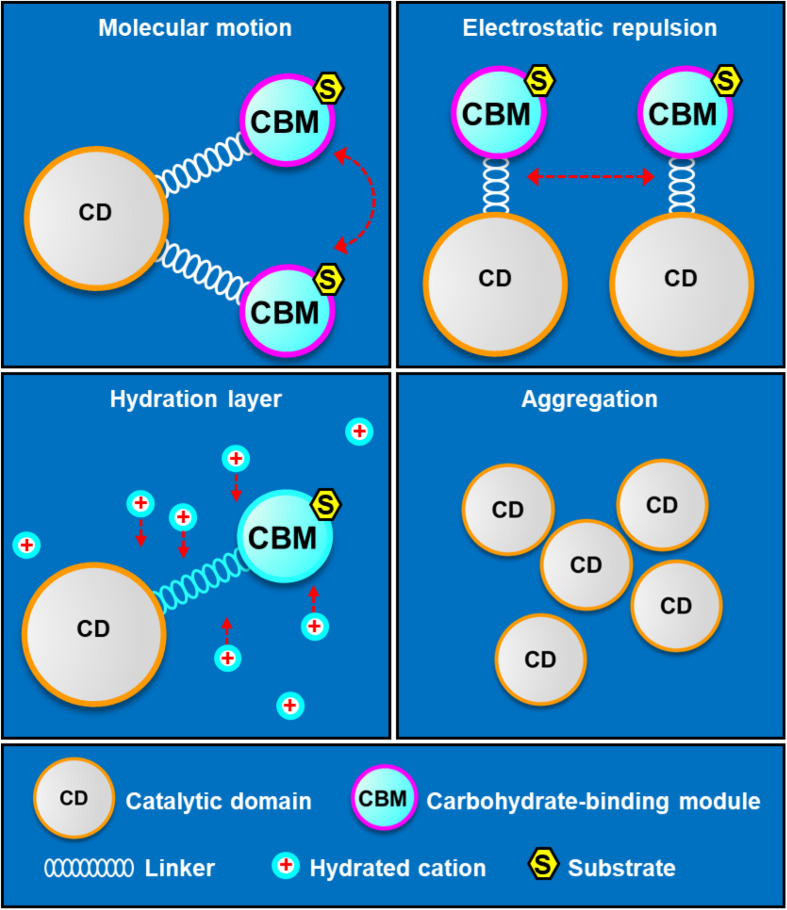
Schematic diagram of structural determinants of cold adaptation and salt tolerance of SdG5A. The highly flexible linker-CBM structure allows an active molecular motion, which guarantees searching and capturing substrates for catalytic domain. The high density of identically charged residues in the linker-CBM region allows appreciable electrostatic repulsion, which prevents protein aggregation and inactivation. The acidic residue-rich linker-CBM structure allows the formation of hydration layer, which help protein maintain in soluble state.

The mechanism of linker-CBM allowing cold adaptation and salt tolerance of SdG5A was summarized in Figure 8. First, the highly flexible linker-CBM structure in SdG5A has evolved to actively search and capture substrates to supply them to the catalytic core when water activity is perturbed by low temperature and/or high salinity. Second, the increased negative potential of the linker-CBM allows electrostatic repulsion between protein molecules, thus preventing them from aggregating. Third, the abundant acidic residues in the linker-CBM help improve the water-binding ability/solubility of SdG5A by binding hydrated cations and maintaining a stable hydration layer. In the case of the non-extremophilic counterpart of SdG5A (SdG5A-CAD), it may tend to aggregate and have lower activity due to the absence of sufficient hydration and mobility initially supported by the linker-CBM structure (Figure 8).

## Conclusion

A cold-adapted and salt-tolerant G5A from marine bacterium Sde 2-40 was expressed in *B. subtilis* and demonstrated to be highly specific for producing G5. Bioinformatics analysis combined with experiments suggested the favorable cold adaptation and salt tolerance of SdG5A might be driven by the increased negative charges and flexible molecular motion of the linker-CBM region. Together, these properties indicated that SdG5A has great potential for both basic research and industrial applications.

## Data Availability Statement

The datasets presented in this study can be found in online repositories. The names of the repository/repositories and accession number(s) can be found in the article/Supplementary Material.

## Author Contributions

ND, ZG, and ZL designed the study. ND, BZ, and BV performed protein modeling and structure analysis. All authors wrote and edited the manuscript and have given approval to the final version of the manuscript.

## Conflict of Interest

The authors declare that the research was conducted in the absence of any commercial or financial relationships that could be construed as a potential conflict of interest.

## Footnotes

1 http://raptorx.uchicago.edu

2 https://zhanglab.ccmb.med.umich.edu/ResQ/

3 https://www.ebi.ac.uk/Tools/msa/clustalo/

4 https://www.ncbi.nlm.nih.gov/cdd

## Abbreviations

AHA: α -amylases from *Alteromonas haloplanctis*
BraG3A: maltotriose-forming amylase from *Brachybacterium* sp. LB25
CBM: carbohydrate-binding module
CorG6A: maltohexaose-forming amylase from *Corallococcus* sp. EGB
DNS: 3,5-dinitrosalicylic acid
G3A: maltotriose-forming amylase
G4A: maltotetraose-forming amylase
G5: maltopentaose
G5A: maltopentaose-forming amylase
G6A: maltohexaose-forming amylase
HPAEC: high-performance anion-exchange chromatographic
KitG3A: maltotriose-forming amylase from *Kitasatospora* sp. MK-1785
MFA: maltooligosaccharide-forming amylase
PAD: pulsed amperometry detector
PseG5A: maltopentaose-forming amylase from *Pseudomonas* sp. KO-8940
PstG4A: maltotetraose-forming amylase from *Pseudomonas stutzeri* MO-19
Sde 2-40: *Saccharophagus degradans* 2-40^*T*^
SdG5A: maltopentaose-forming amylase from *Saccharophagus degradans* 2-40^*T*^
SDS-PAGE: sodium dodecyl sulfate-polyacrylamide gel electrophoresis
ZPA: α -amylases from *Zunongwangia profunda*.
