# Supplementary material for: Carbohydrate-Binding Module and Linker Allow Cold Adaptation and Salt Tolerance of Maltopentaose-Forming Amylase From Marine Bacterium Saccharophagus degradans 2-40T
Source: Front Microbiol. 2021 Jul 14;12:708480. doi: 10.3389/fmicb.2021.708480 (PMC8317173; doi:10.3389/fmicb.2021.708480)
Supplement: Supplementary file 1 [file Table_1.docx]

Supplementary Material

# Supplementary Tables

**Supplementary Table 1 Amino acid composition for catalytic domain, linker and carbohydrate-binding module of SdG5A.**

| Residues | Solvent accessibility ^a^ | Catalytic domain | Accessory domain | Linker | Carbohydrate-binding module |
| --- | --- | --- | --- | --- | --- |
| Asp | E | 6 (1.8%) | 2 (2.2%) | 3 (16.7%) | 3 (3.1%) |
|  | B | 12 (3.6%) | 3 (3.2%) | 0 (0%) | 0 (0%) |
| Glu | E | 4 (1.2%) | 1 (1.1%) | 2 (11.1%) | 4 (4.1%) |
|  | B | 11 (3.3%) | 1 (1.1%) | 0 (0%) | 2 (2.1%) |
| Arg | E | 7 (2.1%) | 5 (5.4%) | 0 (0%) | 1 (1.0%) |
|  | B | 7 (2.1%) | 0 (0%) | 0 (0%) | 0 (0%) |
| His | E | 3 (0.9%) | 0 (0%) | 0 (0%) | 1 (1.0%) |
|  | B | 9 (2.7%) | 1 (1.1%) | 0 (0%) | 0 (0%) |
| Lys | E | 7 (2.1%) | 1 (1.1%) | 1 (5.6%) | 2 (2.1%) |
|  | B | 6 (1.8%) | 1 (1.1%) | 0 (0%) | 1 (1.0%) |
| Ser | E | 8 (2.4%) | 3 (3.2%) | 0 (0%) | 5 (5.2%) |
|  | B | 12 (3.6%) | 1 (1.1%) | 0 (0%) | 2 (2.1%) |
| Thr | E | 4 (1.2%) | 3 (3.2%) | 3 (16.7%) | 9 (9.3%) |
|  | B | 12 (3.6%) | 2 (2.2%) | 0 (0%) | 4 (4.1%) |
| Asn | E | 12 (3.6%) | 10 (10.8%) | 1 (5.6%) | 5 (5.2%) |
|  | B | 12 (3.6%) | 5 (5.4%) | 0 (0%) | 5 (5.2%) |
| Gln | E | 7 (2.1%) | 1 (1.1%) | 0 (0%) | 6 (6.2%) |
|  | B | 8 (2.4%) | 1 (1.1%) | 0 (0%) | 0 (0%) |
| Cys | E | 0 (0%) | 1 (1.1%) | 4 (22.2%) | 0 (0%) |
|  | B | 6 (1.8%) | 1 (1.1%) | 0 (0%) | 2 (2.1%) |
| Gly | E | 10 (3.0%) | 7 (7.5%) | 1 (5.6%) | 6 (6.2%) |
|  | B | 17 (5.1%) | 6 (6.5%) | 0 (0%) | 4 (4.1%) |
| Pro | E | 5 (1.5%) | 1 (1.1%) | 1 (5.6%) | 4 (4.1%) |
|  | B | 13 (3.9%) | 0 (0%) | 0 (0%) | 0 (0%) |
| Ala | E | 9 (2.7%) | 6 (6.5%) | 0 (0%) | 4 (4.1%) |
|  | B | 22 (6.6%) | 3 (3.2) | 0 (0%) | 2 (2.1%) |
| Val | E | 0 (0%) | 0 (0) | 1 (5.6%) | 2 (2.1%) |
|  | B | 25 (7.5%) | 6 (6.5) | 0 (0%) | 5 (5.2%) |
| Ile | E | 0 (0%) | 2 (2.2) | 1 (5.6%) | 0 (0%) |
|  | B | 17 (5.1%) | 6 (6.5) | 0 (0%) | 2 (2.1%) |
| Leu | E | 0 (0%) | 1 (1.1) | 0 (0%) | 0 (0%) |
|  | B | 13 (3.9%) | 0 (0) | 0 (0%) | 4 (4.1%) |
| Met | E | 0 (0%) | 0 (0) | 0 (0%) | 0 (0%) |
|  | B | 6 (1.8%) | 2 (2.2) | 0 (0%) | 0 (0%) |
| Phe | E | 0 (0%) | 1 (1.1) | 0 (0%) | 1 (1.0%) |
|  | B | 15 (4.5%) | 2 (2.2) | 0 (0%) | 2 (2.1%) |
| Tyr | E | 3 (0.9%) | 3 (3.2) | 0 (0%) | 2 (2.1%) |
|  | B | 16 (4.8%) | 2 (2.2) | 0 (0%) | 2 (2.1%) |
| Typ | E | 0 (0%) | 0 (0) | 0 (0%) | 1 (1.0%) |
|  | B | 10 (3.0%) | 2 (2.2) | 0 (0%) | 4 (4.1%) |

^a^ Solvent accessibility was predicted using ResQ server (Yang et al., 2016). Residues with greater than 25% of exposure to solvent were classified as exposed (E), and all others were buried (B).

**Supplementary Table 2 Comparison of the optimum temperatures, cold adaptation, salt tolerance, starch conversion rates and product specificities of G5As.**

| Properties | *Saccharophagus degradans* 2-40^T^ | *Bacillus licheniformis* NCIB 6346 | *Bacillus megaterium* KSM B-404 | *Bacillus megaterium* VUMB109 | *Bacillus* sp. JAMB-204 |
| --- | --- | --- | --- | --- | --- |
| Optimum temperature  (°C) | 45 | 70–90 | 50 | 93 | 60 |
| Cold adaptation | Cold-adapted | Cold-inactive | Cold-inactive | Cold-inactive | Cold-inactive |
| Salt tolerance | Retain 70% of the initial activity at 3 M salt solution | N/A | N/A | Retain 16% of the initial activity at 100 mM salt solution | N/A |
| Conversion rates | Convert 88% of corn starch at 25 °C | N/A | N/A | Convert 20% of corn starch at 90 °C | N/A |
| Product specificity  (%*)* | 79 | N/A | 54 | 40 | 33 |
| Reference | This study | (Morgan and Priest, 1981) | (Auh et al., 2005) | (Jana et al., 2013) | (Hatada et al., 2006) |

N/A, data not available.

# Supplementary Sequences

**Supplementary Sequence 1 Amino acid sequence of SdG5A.** Catalytic domain, accessory domain, linker and CBM were shown in orange, gray, blue and pink, respectively.

QPRTAFVHLFEWQWNDIASECENVLGPKGYAAVQVSPPQKSVSSSQWWSRYQPVSYAIEGRSGNRNEFASMVSRCKNAGVDIYVDAVINHMAAGGRYYPEVPYYAEHFHTCYGQIDYGNRWQVQNCDLVGLNDLATEQDYVRTKIANYLNDLTNLGVAGFRFDAAKHIPVGDIAAIKAKLIGNPYIFQEVIGAPGEPIQPSEYTYLADVTEFNFTKSVSHFFKGRGPIKELKNIGTWGGWVASADAVTFVANHDNQRQDTNNTITHKDGDNRNTMAHVFMLGWPYGYPKVMSSYEWYDHDQGPPAHGASSCNSGWLCEHRTPAIANMVAFRNFTADEFRVTNYWDNGNNQIAWGRGGKGFVVINMEGNNLTRTFQTGMPAGRYCDIISGNYNASNKSCSGNIIDVDNNGNAVITAYYRNGAAIHVGAIVEDCDGTDCCTNCPTEKVETTFSCSNGSTYWGQSVYVIGNQEELGNWNPAHAVKLDASAYPTWTGTIELTQNTDVEWKCLKRDEQTPSNGVVWQGGANNQFNTGTTNAYPTGSF

**Supplementary Sequence 2 Amino acid sequence of MFA from *Brachybacterium* sp. LB25.** Catalytic domain, accessory domain, linker and CBM were shown in orange, gray, blue and pink, respectively.

GDAILNLFQWTWDSVAAECTSTIGPAGFGYVQVSPPQETIQGTAWWTSYQPVSYKIEGKLGTRAEFAAMVETCDAAGVEVIVDAVINHTTGADGGSGTGTAGTPYGIDDFPGIYGAADFNDCRTDISSYQDRYQVQNCRLLSLQDLRTGSEYVQNTLAGYMNDLLSLGVAGFRIDAAKHIPASDLAAIKAKLSDPNAYWVQEVIGAAGEPVQVSEYTGTGDVHEFDYARQLKSDFDGSIANLQRISNGKLPSAQAGVFVDNHDTERNGETMNETWGAKYVLANGETMNETWGAKYVLANTFMLAYPYGSPSVYSGYTFTDEDAGAPGATATSVPDANCDSDAWTCTQRWTEIQGMVGFYNTVAGTALTNWWDDGGNQIAFGRGDKGFVAINNTDSATTRTYSSSLPAGTYCDVVAASDCSETITVAADGTFSATLPAYGALALHVEKTSGGTTPDPDPGTDPVAGKVSASVHASTVLGQEVRIVGSVPELGGWQPASGVALDASGYPSWTGGVDLPAGTSFEYKYVKVDDSGAVVWESGANRTATVGADGTLALNDTWR

**Supplementary Sequence 3 Amino acid sequence of MFA from *Kitasatospora* sp. MK-1785.** Catalytic domain, accessory domain, linker and CBM were shown in orange, gray, blue and pink, respectively.

LGPKGYGAVQVAPPQESISLPGHPWWEVYQPVSYNLNSRMGTDAQFRAMVTACHDAGVKVYADAVINHMAGTNQSSTTGYGGSAFSPSAYSYSAVPYGKSDFHGSPPCPNSDLGINDWNNVTQVQECQLLQLSDLNTESDYVRGKIAGYLNTLIGAGVDGFRVDAAKHIAQADMGNIVSRLSDTQWGGRPYVYQEIFPGSSGQLAPAAFEGNGSVLEFTYASKLTQQFNGNIANLKTFGPSWGFEPSDKSAVMVTNHDLERDKTTLTYNDGSKYKLAHVFELAWGFGTPQVYAGYRFNNKDDSPPADGNGFVTDVNCGNNTWTCTDRDQGIANMVGWHNATKGQAVANWWDNGSNAIAFSRGSKGWVAINNSGSAVTQTFTTGLAAGTYCDVIHGDLSGSNCTGPTVSVDGSGKATVTVNAGDAVALYTATTGTASPSPSSSPSASASPSPSTSPTNPSGTVAENFNATKTTVTGQNVYLVGSVAQLGSWNPSAAIALSPAGYPVWSGSVQLPANTAFEYKYIVKDASGNVTWESGANHSANSGATGGTLNDSWGTATATGQVTVNFSENRTTVVGQNVYLVGSTAQLGSWNPANALLMSSASYPNWKLTLTLPASTAFEYKYVVKDAAGNVTWESGANRTYTTGASGTATLSD

**Supplementary Sequence 4 Amino acid sequence of MFA from *Pseudomonas stutzeri* MO-19.** Catalytic domain, accessory domain, linker and CBM were shown in orange, gray, blue and pink, respectively.

IILQGFHWNVVREAPNDWYNILRQQAATIAADGFSAIWMPVPWRDFSSWSDGSKSGGGEGYFWHDFNKNGRYGSDAQLRQAASALGGAGVKVLYDVVPNHMNRGYPDKEINLPAGQGFWRNDCADPGNYPNDCDDGDRFIGGDADLNTGHPQVYGMFRDEFTNLRSQYGAGGFRFDFVRGYAPERVNSWMTDSADNSFCVGELWKGPSEYPNWDWRNTASWQQIIKDWSDRAKCPVFDFALKERMQNARSPTGSTPERQSRPAWREVAVTFVDNHDTGYSPGQNGGQHHWALQDGLIRQAYAYILTSPGTPVVYWSHMYDWGYGDFIRQLIQVRRAAGVRADSAISFHSGYSGLVATVSGSQQTLVVALNSDLGNPGQVASGSFSEAVNASNGQVRVWRSGTGSGGGEPGALVSVSFRCDNGATQMGDSVYAVGNVSQLGNWSPAAALRLTDTSGYPTWKGSIALPAGQNEEWKCLIRNEANATQVRQWQGGANNSLTPSEGATTVGRL

**Supplementary Sequence 5 Amino acid sequence of MFA from *Pseudomonas* sp. KO-8940.** Catalytic domain, linker and CBM were shown in orange, blue and pink, respectively.

RTAFVQLFEWKWTDVARECETYLGPKGFAAVQISPPNEHNWVSSGDGAPYPWWMRYQPVSYSLDRSRSGTRAEFQDMVNRCNAAGVGIYVDAVINHMSGGNGGTSSAGRSWSHHNYPGLYGSQDFHHPVCAITNYGDANNVQNCELSGLQDLNTGSSYVRGKISDYLVDLVQMGVKGLRVDAAKHISPTDLGAIIDSVNARTGAARPFWFLEVIGAPGEAVQPSQYFGLGGGQVTVTEFAYGKELYGKFAGGGKLADLQTFGPSWNLMPSSKAIAFVDNHDKQRGHGGGGGYVTYHHGSTYDLANIFMLAWPYGYPALMSATASTRAAATTPATARRTTATARPGGRGTGTPPARVLQPDRGWLGLRAPLARHRQHGGLPQRHRGQLVRQRLVEQRQQPDRLRPRRQGLRRHQQGRYGADAASRPACRPGVLRRDLRRLRQRQLHRHGGDRGCRRPRDAVRTRLWRGGDPRRCAHRRHAAGAAVLSLTFNETADTVWGQNLFVVGNVGALGNWAPAAGAAMTWISGSGSTGQWRATVQLPADTPVQYKYVKKDGAGNVVWESGGNRVVTTPAPGATIAVNDSWK

**Supplementary Sequence 6 Amino acid sequence of MFA from *Corallococcus* sp. EGB.** Catalytic domain, accessory domain, linker and CBM were shown in orange, gray, blue and pink, respectively.

VMIQGFHWNSASAGGWWNTVKNNAATLKAAGFTMIWLPPPSDAASTQGYLPRQLNVLNSSYGTEAELTAALAALNAQGIKPIADIVVNHRVGTTNWADFTNPTWPGCSAVVAGDEWTGACGNADSGEGYAAARDLDHSQANVRADLKTWMNSRLKGVGFAGWRFDFVKGFAGSYVKEYVAATDPWFCVGEFWPTNYFDANNPNDWKQQIVNWVDATTGTCAAFDFATKGLLNDALTNNNYTRLKASDGKPAGGIGWWASRHVTFVDNHDTGPSESCGNGQNHWPVPCAKVMQGYAYVLTHPGIPTVYWAHYFNWGLGSSIKALMDIRKSAGLTSESTVSIQRAESGLYAAIIGGKVAVKLGSGSWSPGTGWTQAASGTDYTVWTTNTPPPTGTTANVEFVCNNGTTVMGQNVYVTGSVAELDTWSPTTTKILSPTAYPTWRGTYALPANTTVQWKCLKRDGSGNVVWQGGSDNTLTTPAAGGSTTATASF

**Supplementary Sequence 7 Amino acid sequence of AHA.**

TPTTFVHLFEWNWQDVAQECEQYLGPKGYAAVQVSPPNEHITGSQWWTRYQPVSYELQSRGGNRAQFIDMVNRCSAAGVDIYVDTLINHMAAGSGTGTAGNSFGNKSFPIYSPQDFHESCTINNSDYGNDRYRVQNCELVGLADLDTASNYVQNTIAAYINDLQAIGVKGFRFDASKHVAASDIQSLMAKVNGSPVVFQEVIDQGGEAVGASEYLSTGLVTEFKYSTELGNTFRNGSLAWLSNFGEGWGFMPSSSAVVFVDNHDNQRGHGGAGNVITFEDGRLYDLANVFMLAYPYGYPKVMSSYDFHGDTDAGGPNVPVHNNGNLECFASNWKCEHRWSYIAGGVDFRNNTADNWAVTNWWDNTNNQISFGRGSSGHMAINKEDSTLTATVQTDMASGQYCNVLKGELSADAKSCSGEVITVNSDGTINLNIGAWDAMAIHKNAKLNTSSASSTESDWQRTVIFINAQTQSGQDMFIRGGIDHAYANANLGRNCQTSNFECAMPIRHNNLKNVTTSPWKANDNYLDWYGIENGQSSEAEGSATDWTTNVWPAGWGAEKTVNTDGFGVTPLNIWGEHYWMLDVDMDCSKAVNGWFELKAFIKNGQGWETAIAQDNAPYTSTNHMAQCGKINKFEFNNSGVVIRSF

**Supplementary Sequence 8 Amino acid sequence of ZPA.**

QIDKMEPPFWWSDMNLEELQIMFYGKNIGTYEVSSEDEVIINNIRKTENPNYVFVTINSSALPAGNYEFNFSKKGKRNITKTFELKQREEGSALREGFDASDAIYLIMPDRFANGNPDNDSSTEMQEKADRSKQGGRHGGDIQGVIDHLDYLKNLGITALWSTPLLADDDAGYSYHTYAQSDVYKIDPRYGSNEDYKRLANELHQKDMKLIMDYVTNHWGSEHWMIKDLPTYDWVHQFPGYENSNYRMTTQYDPHKSARDFKYCVDGWFTGTMPDLNQSNPLVLNYLIQNAIWWIEYSGLDGFRVDTYSYNDKEGIAKWTKAIMDEYPYFNIVGEVWMHDQAQISYWQKDSPIGAIQDYNSNLPSVMDFTLHDAMTSMFHEQDASWDRGMIKAYENFVNDFLYADTDNLMVFMGNHDTGRFNEIYDGDFKKYKMAMTMIATVRGTPQIYYGDEIGMRGDKGKGDGAIRQDFPGGWEGDEQSAFNAEDRTENQMKYFDLTSKLLNFRKENEVLQFGKMLQFLPENNVYVYFRYNDKNRVMVIINNNAEEQTLDLKKYAEGIQGSTSGKEIISGKDIQLNETLSIPAQDAMLIQLQ

**Supplementary Sequence 9 Amino acid sequence of Aga50D.** CBM was shown in pink.

GSHMLFDFENDQVPSNIHFLNARASIETYTGINGEPSKGLKLAMQSKQHSYTGLAIVPEQPWDWSEFTSASLYFDIVSVGDHSTQFYLDVTDQNGAVFTRSIDIPVGKMQSYYAKLSGHDLEVPDSGDVNDLNLASGLRSNPPTWTSDDRQFVWMWGVKNLDLSGIAKISLSVQSAMHDKTVIIDNIRIQPNPPQDENFLVGLVDEFGQNAKVDYKGKIHSLEELHAARDVELAELDGKPMPSRSKFGGWLAGPKLKATGYFRTEKINGKWMLVDPEGYPYFATGLDIIRLSNSSTMTGYDYDQATVAQRSADDVTPEDSKGLMAVSEKSFATRHLASPTRAAMFNWLPDYDHPLANHYNYRRSAHSGPLKRGEAYSFYSANLERKYGETYPGSYLDKWREVTVDRMLNWGFTSLGNWTDPAYYDNNRIPFFANGWVIGDFKTVSSGADFWGAMPDVFDPEFKVRAMETARVVSEEIKNSPWCVGVFIDNQKSFGRPDSDKAQYGIPIHTLGRPSEGVPTRQAFSKLLKAKYKTIAALNNAWGLKLSSWAEFDLGVDVKALPVTDTLRADYSMLLSAYADQYFKVVHGAVEHYMPNHLYLGARFPDWGMPMEVVKAAAKYADVVSYNSYKEGLPKQKWAFLAELDKPSIIGEFHIGAMDHGSYHPGLIHAASQADRGEMYKDYMQSVIDNPYFVGAHWFQYMDSPLTGRAYDGENYNVGFVDVTDTPYQEMVDAAKEVNAKIYTERLGSK

**Supplementary Sequence 10 Amino acid sequence of Xyn10C.** CBM was shown in pink.

MREKLLRALLTSAKFFGASLLLLSLFNLTACGGGSSGTKPVVEEPQPEPQPEPEPEPEPEPEPEPEPEPQPEPEPEPDFSALHTDGTKWVNANGDQVLLKGVNLGNWLLQEFWMMEQGSEDVNDQCSLEAVFDDRFGFAERERLMDLFRDNWINDRDWDIIASFGMNVIRLPFIWNLIEDENNPMTLRDDAWQYIDYAIEQAEARDMYVILDLHGAVGAQGWEHHSGCAELNEYWGSEAYQERTRWLWQQVATRYADRDAVAAYGVLNEPWGTTPENLAVEAIELFDAIREVDADKIIILPGHHSGIHAYPNPATVNQTNVAYEMHFYPGIFGWGEIGYDVNRDWLTCGPTGTSGVCEWDARLDALDSPFLIGEFQPWTGLGPELGAQITRATYDTYASFDWASTAWSYKIITSGGGQGGGTWGMVTNERGLGLLAKADTWACAGWDSSFANACGVSRTGFTPDREGEQTYYLVIKFGACCEGNLDATLDSISIIDDVTGEEIIVNGGFGAGTGWTEWYESAMPIIDYNYTGAGVPTGSDGAVLRMSGAAAINGGVYQAITLDSSKSYSFSGVFKDNGSASAWAEIFLVQSQPVDGSDVLAEGPFAAVDFLTAPIEEIENLFEAFGTTPYDIHEEMRAAMTAETAPTLFDLPGAPTGVMLAEDAGAATISWTASGDANVTGYNVYRSTISGNSYTLLAENVTATTFVDSTIDGEQTFYYTVTAVTDTAESYRSQEVATTFVAVHLPGKVEAEAHSDMMGLQTENTTDTGGGINIGFIDAGDWFEYEVTIDTAATYNIHYRLASEPGSTGFTVSINDEVLNTVAVPATGGWQTWQTESTTITLPAGEHTLRFDALGGQWNMNWWSVEAVD
